# Alcohol education in Ugandan primary schools: teaching approaches and learners’ perspectives

**DOI:** 10.1186/s12889-025-23043-1

**Published:** 2025-05-17

**Authors:** Neda Valeckaite, Melf-Jakob Kühl, Juliet N. Babirye, Aisha K. Kiirya, Ingunn M. S. Engebretsen

**Affiliations:** 1 Centre for Internation Health, Department of Global Public Health and Primary Care, University of Bergen, Bergen, Norway; 2 School of Public Health, Collage of Health Sciences, Makerere University, Kampala, Uganda

## Abstract

**Background:**

Alcohol use is a major health concern in Uganda, affecting children and adolescents directly through their own consumption or indirectly through other’s drinking. Schools have an opportunity to improve knowledge and address attitudes towards alcohol. While curriculum-based alcohol education is a widely used educational approach, there is limited research on its delivery and effectiveness in Ugandan primary schools. This study explored teachers’ and learners’ perspectives on alcohol education in Ugandan primary schools, focusing on teaching methods and key content areas.

**Methods:**

This qualitative study was conducted in six purposively sampled primary schools in Mbale, Eastern Uganda. We conducted two focus group discussions in each school, a total of twelve, with 7–8 learners per group, aged 11–13 and a total of twelve key informant interviews with the teachers. The data was coded in NVivo and analysed using reflexive thematic analysis.

**Results:**

Alcohol education was primarily integrated into science lessons in Ugandan primary schools. Four main topics were covered during the lessons: what alcohol is and how it is made; the effects of alcohol use; why people drink; and, how to avoid it. The dangers of alcohol use were strongly emphasized by the teachers and the children were advised to avoid places and people including children that use alcohol. Teachers often used moralising and didactic methods of teaching to underline their message against alcohol use.

**Conclusion:**

This study shows that teachers considered alcohol education as an important topic aiming to teach children about the dangers and negative effects of alcohol use.

**Supplementary Information:**

The online version contains supplementary material available at 10.1186/s12889-025-23043-1.

## Introduction

Alcohol use is a major disease contributor, especially, for the youth population where mental and substance use disorders are the leading cause of disability [1–4]. Alcohol consumption before the age of 14 is associated with adverse consequences such as alcohol dependency later in life [1]. Due to lower access to health services and social security, alcohol use is relatively more harmful in low-income countries compared to high-income settings [1]. The Ugandan population consumes on average 12.2 L of pure alcohol per capita, twice the global average [5]. Many households rely on alcohol production as a source of income, making homebrewed alcoholic beverages readily available and affordable in the region, with limited implementation of regulations in place [6–8]. Common alcohol consumption in the community and lack of implementation of effective alcohol control policies make children vulnerable to harmful alcohol use [2, 8–10]. Studies report that in Uganda some children are exposed to alcohol at an early age by their parents and the community [6, 11, 12]. One study found that 7.4% of 5–8 year-old home welling children with mental health symptoms also reported alcohol abuse and dependence [13].

Adolescence is associated with increased risk-taking behaviour including alcohol use, thus the primary school setting is especially relevant for raising awareness and reducing alcohol related harm [14, 15]. Studies show that school attendance, connectedness to school staff and a positive school environment are protective factors against alcohol use [16, 17]. Conversely, school drop-out is a significant risk factor associated with early alcohol use in Uganda [18]. The introduction of universal primary education in 1997 resulted in a considerable increase in primary school enrolment in Uganda, however, the drop-out and class repetition rates remain high [19, 20]. The Uganda School Health Policy from 2008 emphasizes the role of schools in promoting a healthy psychosocial environment, yet the prevalence of alcohol and substance use continues to be relatively high [21–23]. A cross-sectional study including adolescents and young adults aged 12–24 years found that 70% of secondary school learners had tried substance and almost 2 out of 5 regularly used them with alcohol being the most common one [23]. Another study from eastern Uganda reported that 28% of primary school learners aged 10–13 had consumed alcohol in the last year [24].

To date, only a few school-based interventions focusing on alcohol have been conducted and assessed in Africa [14, 25, 26]. In Uganda, previous qualitative studies have described the child and adult perspectives on alcohol use, and some studies have explored the teaching of the standard primary curriculum [11, 13, 27–30]. Although, the Ugandan primary school curriculum incorporates alcohol education, there remains limited research on the impact of these educational initiatives in shaping learners’ knowledge, attitudes, and behaviors regarding alcohol use. Furthermore, there is a lack of insight into learners’ perceptions of alcohol education, how it aligns with their lived experiences, and the factors that influence their attitudes towards alcohol use. This gap in knowledge undermines efforts to create meaningful and impactful alcohol education programs that resonate with children and contribute to reducing early alcohol use initiation. Therefore, this study aimed to explore the teachers’ and learners’ perspectives on alcohol education and teaching methods used in Ugandan primary schools.

## Methods

### Study design

We conducted a qualitative study to explore teachers’ and learners’ experiences and perceptions on alcohol education. Data was collected using key informant interviews (KIIs) with teachers and focus group discussions (FGDs) with learners. The semi-structured KII (Supplementary file 1) and FGD (Supplementary file 2) guides were developed specifically for this study by the researchers. With this design, we aimed to explore separately, both the learners’ and teachers’ perspectives on alcohol education. We opted for KIIs to allow for an in-depth exploration of teachers’ experiences in their school and to facilitate more open responses in cases of sensitive subjects. FGDs could capture learners’ collective opinions and experiences with alcohol education, while offering the children a potentially comfortable and safe setting among peers.

#### Study setting

Data was collected from May to July 2022 in Mbale District, Eastern Uganda. Uganda is considered a low-income country with 42.2% of the population living on $2.15 or less per day [31]. The country has 47 million inhabitants where half of the population is under 15 years of age [31]. Primary school education comprises seven grades, primary one (P.1) to primary seven (P.7). In Mbale, classrooms are often crowded with an average of 77 children per classroom [32].

#### Current curriculum

The primary 6 (sixth year of formal education) national science curriculum guidelines state that: “In this topic, you should guide the learners on the dangers of smoking, alcoholism and drug dependence.” [33]. The curriculum outlines the content for the lesson plan including the following topics; what is alcohol, how it is made, factors that lead to alcoholism and how alcoholism affects the individual, family and the community [33]. Additionally, it provides examples of teaching methods such as dramatizing, brain-storming and experiments that can be used during the lessons [33]. The key message that should be conveyed is that substance use is harmful to their body therefore they should acquire life skills to avoid it [33].

#### School sampling

We purposively sampled six out of 131 public primary schools in Mbale [34]. Eighty-four schools were found to be registered on Google Maps and within one hour driving distance from Mbale city. We restricted our sample to three schools within 15 km of the city centre and three schools outside the 15 km range. We further aimed to capture diverse views from different populations, thus further narrowing the criteria to two schools from rural, peri-urban and urban areas, respectively. While all selected schools enrolled students from varying religious backgrounds, we purposefully included one Islamic faith school, while the rest were Christian based schools. Four of the schools were day schools while two enrolled both day and boarding learners. All schools had female and male learners.

#### Participants for the key informant interviews

Headmasters from the six sampled schools were initially informed of the planned study and asked to participate. All approached schools agreed to participate in the study. The headmasters identified the science teachers who taught science lessons in the school. We recruited 12 science teachers, one to three teachers from each school. All teachers were teaching health topics in the school for the Primary 4 to Primary 7 grades (Table 1). All teachers gave written informed consent to participate before we conducted the 12 KIIs. The date and time for the KIIs was agreed upon the first school visit. The interviews were conducted in English during school hours on school premisses by the first author (NV) supported by a local research assistant.

#### Participants for the focus group discussions

We also conducted 12 focus group discussions (FGDs) with the learners, one group with girls and one group with boys per school. We stratified the groups based on gender to ensure homogeneity within the groups and to facilitate openness. Each group included 7–8 participants aged 11–13 years, attending grades from Primary 3 to Primary 7 (Table 1). In total, 92 learners participated in the FGDs, with an equal gender balance (Table 1). To identify suitable research participants the research team collaborated closely with the science teachers. The purpose of the study and the inclusion criteria of the learners were carefully explained to the teachers after which they were asked to identify learners who fit the age criteria, were not related and would be comfortable with speaking to the researchers. After the inclusion the research team dismissed one child as he was feeling unwell during the FGD. The first author was present during two FGDs in an urban school since the urban youth population was more comfortable expressing themselves in English. The rest of FGDs (*n* = 10) were conducted by the two research assistants in the language preferred by the group, including Lumasaba, Luganda and English, the three languages are commonly used in the Mbale area.

Data was collected by a team of three Ugandan research assistants and the fist author (NV). At the time of data collection, the fist author was a third-year medical student and enrolled in The Medical Student Research Program at the University of Bergen, Norway. Two research assistants, who were experienced in collecting data from minors and spoke the local languages conducted the FGDs with the learners. One research assistant held a bachelor’s degree in social science, while the other had a diploma in social work. KIIs were conducted by AKK who has a bachelor’s degree in education and NV. The data collection was iteratively adjusted based on interim results from each school visit. We considered the 12 KIIs and the 12 FDGs from six schools with diverse characteristics sufficient to capture a wide spectrum of practices and perceptions. Additionally, as the last few interviews and discussions yielded limited new information, we determined that the saturation was met.

We use the terms “learner” and “child” interchangeably for the FGD participants. “Learner” describes the population characteristics, while “child” was widely used by their teachers.

#### Procedures

Semi-structured guides were used for the KIIs and the FGDs. The participants were asked open-ended questions about their education on general health, mental health, and alcohol and substance use. The interview guide was pre-tested by the research team and the focus group discussion guide was pre-tested with Ugandan primary school learners among schools that were not selected for study participation. The KIIs lasted between 40 and 80 min while the FGDs took 50 to 100 min. Audio recorders were used to record the FGDs and KIIs. All KIIs were transcribed verbatim by the fist author. During FGDs the participants were encouraged to use their preferred language to express themselves more freely. FGDs were transcribed verbatim and translated by two research team members who also conducted the interviews. After transcribing, the audio files were rerun, and the transcripts were double checked.

### Analysis

The first author followed the Thematic reflexive analysis guide outlined by Braun and Clarke [35]. Data transcripts were repeatedly read to gain a deeper understanding of the data set. For each KII and FGD the first author wrote a short summary and notated interesting aspects of the data. After the familiarization of the data the first author utilized qualitative data analysis software NVivo12 for open coding of the data. The codes were kept separate for the KIIs and FGDs for better clarity of the different datasets and because we were interested in identifying possible differences between the perceptions of the teacher and the learners. A spiral approach was taken during theme generation where codes where revised and re-clustered after developing the initial themes [35]. The initial themes were developed in Microsoft Word using the coded system from NVivo and combining both participant codes. Themes were discussed within the research group and further developed, refined, and recombined. Quotes were then selected based on their ability to illustrate a theme. We aimed to represent different participants, capturing both teachers’ and learners’ perspectives and different categories of rural, peri-urban and urban school affiliation.

### Validation

In December 2023 six teachers from the participating schools were invited to take part in a presentation of preliminary research results and to express whether their perspectives were appropriately reflected. At this instance, they were also given an opportunity to correct preliminary results and to adjust the importance ascribed to them by the research team.

## Results

In this article we describe the teachers’ approaches to educate the learners about alcohol use. Thus, the focus is on the teachers’ description of alcohol education while the learners’ perspectives are used to triangulate the findings. We use teachers interviews to describe alcohol education and the teaching methods while learners’ perspectives provide nuances on how the lessons are received.

We identified two main themes on alcohol education in Ugandan schools. The first one *“Teaching and learning about alcohol”* which includes the following sub-categories; (a) how alcohol is made, (b) the effects of alcohol use and (c) reasons for drinking it. The second theme *“Strategies to deter learners from using alcohol”* explores the advice and methods used to deter learners from alcohol use. For each theme, we bring in both perspectives from the learners and the teachers.

Table 1 gives an overview of the characteristics of the participants. The majority of the learners were aged 12 and above and in Primary 6 and Primary 7 levels. It was an even mix of boys and girls, but all the teachers were male, and the majority had a 2-year diploma.

**Table 1:** Presents an overview of participant characteristics for the FGDs and the KIIs

**Focus group discussions**		**Key informant interviews**	
**Total participants**	92	**Total participants**	12
**Gender**		**Gender**	
Female	46	Female	0
Male	46	Male	12
**Age**		**Teaching experience**	
11 years	16	10–20 years	7
12 years	28	> 21 years	5
13 years	48		
**Grade**		**Education**	
Primary 3–5	28	Diploma 2 years	9
Primary 6–7	64	Diploma 4 years	1
		Bachelor’s degree 3 years	2
**Place of schooling**		**Place of employment**	
Rural	29	Rural	3
Peri-Urban	31	Peri-Urban	5
Urban	32	Urban	4

### Teaching and learning about alcohol

#### How alcohol is made

Children learned about the benefits and disadvantages of alcohol during science class. Both teachers and children described alcohol as a source of income for the producer and a cause of poverty for the consumer. Both agreed that it was dangerous for the children to be in the household where alcohol was prepared, but they also acknowledged that families were dependent on the income from alcohol production to provide basic needs and tuition for the children. *“[It] becomes a problem*, *because now the parent is selling alcohol in order to earn a living. So*, *these children are always around that alcohol. That one is a big challenge.” (Teacher*, *Urban)*. Teachers reasoned that it was important to teach learners the process of alcohol production. Therefore, practical experiments during science lessons included showing the process of distillation of alcohol. *“We teach them because in the future this alcohol is a business.” (Teacher*, *Rural)*. Teachers were aware of learners that lived in families where alcohol was brewed and noticed that some were already familiar with the process of alcohol preparation. “*Others even know how to prepare it because they come from these communities where they prepare.” (Teacher*, *Urban).*

#### The effects of substance use

Education on substance use was centered around teaching the consequences of alcohol use to the individual, family, and community. The individual effects included damage to the organs such as the kidneys, the intestinal tract, and especially the brain. Alcohol was said to affect the physiological wellbeing of the brain as well as cause mental health issues. “*Alcohol can destroy the brain and the mental health is destroyed. The person develops a mental disability and that can make you run mad.” (Boy*, *Urban)*. Both teachers and children expressed that alcohol could affect the reasoning capabilities, thus people under the influence of alcohol could hurt themselves and commit crimes within the community. “…*the teachers told us that a person who is drunk or is under the influence of alcohol does not understand what he or she is doing. That’s when some of them*, *when they are drunk*, *can do other things like stealing*, *raping young girls and others.” (Girl*, *Peri-Urban).* Some children implied that alcohol controlled people, thus a drunk person could not account for their actions. *“When you take alcohol and get drunk it commands you to go and beat a child…” (Boy*, *Rural).*

Learners highlighted many dangers to children and other family members when alcohol was consumed in a household. Children differentiated between the consequences if a mother or a father was drinking. If a mother was drinking it would lead to child neglect and lack of prepared food at home. Similarly, drunk fathers were also associated with lack of basic needs and school fees. However, more importantly drunk fathers inflicted domestic violence. *“For me the challenge which I have seen with alcohol*, *it’s the men who mostly use it and if taken in excess can reach home drunk and starts beating the mother and the children because he will have lost his memory.” (Girl*, *Urban)*. In general, the learners thought that alcohol use would cause family separation where the children would need to provide for themselves for their survival. *“I feel bad because*, *where I stay*, *the parents who drink and most of my friends come from there…sometimes these children pick scraps to go and sell so as to get money to get food and others may end up getting the leftover food so that they can eat or survive.” (Boy*, *Urban).*

The children were also taught the consequences of alcohol use to the community. Both teachers and children described alcohol users as criminals who were violent and committed theft to be able to afford alcohol. “*They tend to beat people who pass by them and yet the people they beat are innocent.” (Girl*, *Rural).*

#### Reasons why children drink

Teachers and children noted several reasons for why children drink alcohol. Teachers expressed that a child learned by copying others therefore they considered parents as one of the key factors initiating children to alcohol use. The influence from parents was highlighted by a teacher who described a drinking place near the school where the parents of the learners would go to drink while their children were at school. *“Alcohol is being used*, *there are very many drinking joints. So*, *as they drink from there you never know*, *children may copy their vice… Yeah*, *these are parent. Almost every home has a child here.” (Teacher*, *Rural)*.

Both teachers and children also identified peer pressure as a key factor for alcohol use by children. “Bad peers” encouraged, or in some cases, forced other children to drink. Peers could exert pressure to use alcohol by speaking positively about alcohol or by threatening exclusion from the friend group. *“Us children sometimes we can be influenced by other friends: “If you don’t drink alcohol*, *don’t walk with me” -so*, *you just have to take because you want to be their friend.” (Girl*, *Urban)* In addition, alcohol use could provide a higher status within the group where children used alcohol to seem more mature, to gain confidence to express themselves and to show that they could afford alcohol. *“So that maybe ‘let me do so that I can fit in the group. Let me show my friends that I have also matured.’ Others are shy*, *cannot express themselves freely. They feel when you take alcohol*, *you pick that confidence.” (Teacher*, *Urban).* Teachers reported that peer pressure was exercised by older learners in secondary schools or by children who had left school. One teacher described friend groups enticing learners to miss class. *“In this community here*, *we have boys who are school dropouts… when you are teaching you find them coming…you find out that they want some of the children in class who are their colleagues… Some of them are absent in class*, *but in the evening*, *you will see them passing with those group mates” (Teacher*, *Peri-Urban)*.

### Strategies to deter learners from using alcohol

#### How to avoid drinking?

The learners were taught to avoid places and peers that used alcohol to safeguard themselves from the negative effects of drinking alcohol. Teachers warned children about cinemas and discos as well as advised against TV and media as they were deemed to send the wrong messages to the learners, teaching them “bad” behaviors. *“We talk about that children shouldn’t take alcohol*, *don’t go to discos*, *don’t go to films because when you go there they take up the new things which they learn and get spoiled.” (Teacher*, *Peri-Urban)*. Teachers also advised learners to avoid people that use substances because they could be dangerous and violent. Moreover, teachers advised to leave bad friend groups as children’s mindset towards alcohol could be influenced by the collective behavior and values of the group they were in. *“So even you go counseling them*, *telling them always try to move with a good group*, *leave the bad group. It is like theft. If you move with a person who steals*, *you will also steal. That is what we keep on telling.” (Teacher*, *Rural)*.

Learners expressed concern for peers who used alcohol as it could ‘spoil’ their future, lead to school dropout and homelessness among other negative consequences. They would encourage a friend to stop drinking by advising them on the dangers of alcohol, however also expressed willingness to distance themselves from that friend.*“…I will tell him that*, *‘Do you remember what the teacher told us about the bad and good things of alcohol?’*, *then I tell him that do you know that alcohol affects our body*, *if he refuses then I separate myself from him.” (Boy*, *Peri-Urban).* Furthermore, they did not want to be associated with alcohol users in the community. The learners were worried that the friend would lose respect in the community due to their behavior which in turn would reflect negatively on the learners themselves. *“I would feel ashamed because if my own friend is drinking and using those other substances*, *people will begin to suspect that I also take alcohol.” (Boy*, *Peri-Urban)* Lastly, children were concerned that their friend could influence them to use alcohol, thus many reasoned that it was safer to avoid that friend. *“For me I just avoid walking with that friend of mine. It’s because he may also teach me how to drink alcohol and those other substances.” (Boy*, *Rural).*

Some learners thought that children who used substances should be expelled from the school or arrested by the police so that they would not ruin the school environment or the community. *“I think they should be chased away from school because he is teaching other children bad behavior.” (Girl*, *Peri-Urban)*. Teachers could also employ harsh punishments for the learners who were caught using substances. Punishments included caning and expelling learners from the school. These punishments signaled clear standards against alcohol use and were used against the recurrence of alcohol use by the child and as deterrents for other learners. Additionally, expelling a misbehaving child was done for the purpose of safeguarding other learners against alcohol use. *“Because we look at the over 700 children we have*, *and you*, *we have counselled you three*, *four times and you have insisted. We can’t leave you then to spoil the rest. We kick you out*, *call the parent officially*, *go.” (Teacher*, *Peri-Urban)*.

#### Emphasizing the dangers

Since learners were taught about the negative effects of alcohol use, the teachers used these effects to deter alcohol use. They used strong vocabulary such as “destroy” and “damage” and could imply that these effects were inevitable for the alcohol user. “*…you will have to damage the brain*, *it is a must. Two*, *you will also have self-neglect*, *you will not bathe*, *you will not do anything. Then it will lead to poverty*, *you are not going to work…as you take a lot you will get a lot of other diseases. For example*, *the heart*, *the lungs they will be destroyed*, *because it has a lot of toxins. (Teacher*, *Rural)* The effects of alcohol could also be exaggerated where even tasting alcohol could lead to addiction. *“Say no to alcohol because it is addictive. If you start taking small quantities*, *they will end up being addicted. That’s what we teach on alcohol.” (Teacher*, *Peri-Urban).*

Teachers used stories from the community to emphasize the negative effects of alcohol, sometimes even giving learners’ parents as examples. *“We teach them the dangers of alcohol… Then*, *from there*, *you say: ‘alcohol is bad. When you look at your parents their health is not that good. Some of them died before their time comes. Their lifespan is short*, *those people who take alcohol.’” (Teacher*, *Urban)*. Teachers used negatively charged narratives depicting drunk people to create an unflattering image of an intoxicated person and prompted children to imagine themselves in that situation. *“’How do you look at these people who are taking more of alcohol in your community? Do you admire looking at them? Do you admire what they do?’ They really tell you no. So*, *you really see they can learn something out of it. Cause like neglect*, *there are some people who are neglected*, *so you ask them*, *‘do you wish to be in such a situation?’” (Teacher*, *Urban)*.

Teachers thought that knowledge about the dangers of alcohol could dissuade children from substance use and ensure they did not develop positive associations with alcohol. They aimed to empower learners to make the ‘right’ decisions regarding alcohol use. *“The dangers. We talk about the dangers. We emphasize more of the dangers. Because you cannot tell them that alcohol is good. Cause for them*, *they are still young*, *they cannot control. (Teacher*, *Urban)*. Children also valued learning about the negative effects and reasoned that knowing the dangers prevented them from drinking alcohol. *“The topic which was interesting was that of alcohol because it helps me to know the dangers of alcohol and also to advise people to stop drinking because it may harm their brain and in case you have a father who drinks*, *you should tell him to stop drinking alcohol.” (Girl*, *Urban).* Furthermore, explaining the dangers and giving examples of people struggling was seen as effective because the children could observe the consequences of alcohol use in the community. *“…they do take for my case*, *they do take [my advice] because when we talk about the effect of alcohol it is there in the communities*, *they even know the alcoholics. And they know how these people are suffering where they are.” (Teacher*, *Peri-Urban).*

As a consequences of this danger focused rhetoric the learners expressed fear of severe consequences of substance use which included violence, stealing, rape and death. They were worried about getting sick or dying because of alcohol use. Death from substance use was caused by accidents on the road, getting into fights while drunk or due to the alcohol itself. *“If I see my friend with the substance and alcohol*, *I feel bad because it leads him to die*, *and I will have no friend.” (Boy*, *Peri-Urban)*. Young girls were seen as especially vulnerable to rape if they used alcohol or were in proximity of intoxicated people. One focus group expressed that people drink alcohol so they could rape children. *“They drink alcohol to hurt other children like raping them. Yes. People take alcohol to rape children.” (Girl*, *Peri-Urban).* A few girls also said that they were being taught to stay away from older men, including drunk fathers, to safeguard themselves from rape. *“They taught us that for girls who have reached the adolescent stage*, *you should avoid being near older men like boda-boda cyclist…and if your father drinks alcohol*, *you should avoid being near him because he may get drunk and end up raping you.” (Girl*, *Urban).*

Although teachers mostly expressed teaching the dangers of substance use, children shared that alcohol could be beneficial and used positively in the community. Learners explained that alcohol was used to feel strong and get more energy for hard labor such as digging or stone quiring. They thought that people drank because of boredom, to relieve stress, solve their problems and to sleep better. Alcohol was also used during social occasions such as circumcision ceremonies to feel happy and socialize with others. *“It creates unity. Like when you are taking local brew it creates unity because of drinking from the same pot.” (Boy*, *Urban)* Teachers were aware that children had positive associations with alcohol, and some explained that they addressed these ideas in class, calling them misconceptions.

Teachers also acknowledged that not all learners were receptive to their danger focused rhetoric on substance use. They explained that some children had a positive outlook on alcohol. It was difficult to teach those learners the negative effect of alcohol. Teachers explained that it was impossible for them to reach every child and their role was to insist on the dangers of alcohol use and provide learners with the right knowledge. *“We cannot remove these learners totally from knowing. They know them*, *they are trying to test. But for us as teachers*, *we teach them the dangers…They have bad peer-groups in the community who are taking that thing. So when we talk about it badly*, *… they think you are just preventing them to take the good thing…so that is why you need to keep on insisting ‘Don’t take*, *these are the problems.’” (Teacher*, *Rural)*.

Lastly, although most learners thought that alcohol was dangerous, there was also a wide range of opinions on the safe amount of alcohol to consume. The amount varied from nothing at all, to a bottle, half a bottle or only one cup per one sitting. Furthermore, their view of how often alcohol could be consumed safely varied likewise. Some thought that alcohol should be taken once a month, once a week, every other day and even daily. Learners also had different opinions of when alcohol should be consumed where some thought that alcohol should only be used during celebrations while others said that you should drink before you sleep, if you are stressed or if you have problems. *“We should take little like 1/2 a bottle in a day and then take after 2 days again.” (Girl*, *Urban)*.

## Discussion

Teachers participating in this study adhered to the science curriculum and covered alcohol-related topics in class. They concentrated on four principal facets of alcohol education: production of alcohol, reasons for taking alcohol, effects of alcohol, and how to avoid it. Two predominant strategies were used to deter children from alcohol use, namely, the avoidance of places and peers that consumed alcohol, as well as the danger-focused rhetoric aiming to provide knowledge about the dangers of alcohol and create negative attitudes towards it. As a result, most children perceived alcohol as a dangerous substance that harms the body and negatively impacts communities, families and peers. Similarly to their teachers, they believed that the best way to safeguard themselves from alcohol use was to avoid people and places associated with it.

### Avoidance of alcohol

Science teachers followed the universal educational curriculum on alcohol use for 6th graders and among them they employed similar methods of teaching children about alcohol use. The effectiveness of school-based alcohol programs varies: most interventions increase knowledge and change attitudes towards alcohol but demonstrate limited impact on actual drinking behavior [36–39]. Alcohol education through classroom curriculum is a widely used approach to educate learners about alcohol and to prevent alcohol use [40]. However, there is little research on the delivery and the effectiveness of the Ugadan primary school alcohol education curriculum and overall, few school-based alcohol interventions have been conducted and assessed in Africa [25, 39]. Alcohol use is a complex issue where personal characteristics and the environment influence person’s beliefs, attitudes, and behaviour towards alcohol [9, 38, 41]. Although no definitive approach has been proven to lower alcohol use in different school settings, some educational approaches appear more effective compared to others.

Grube’s and Morgan’s theory suggests that a positive attitude towards alcohol is more likely to manifest if the environment supports it [42]. Schools are a key source of alcohol knowledge and can shape learners attitudes towards substance use [43]. As such, school can have both a conducive and a deterring role with respect to child alcohol use. The participating teachers in this study aimed to project a clear value of “zero tolerance” to alcohol and focused on creating a disapproving environment by teaching the disadvantages and negative aspects of drinking and by encouraging children to avoid alcohol. Teachers’ disapproving attitude towards alcohol use was also underlined by verbal or physical punishment of children who consumed alcohol. There is merit to suggest that this approach, to categorically adopt a disapproving position against alcohol and alcohol users, could limit substance use by the learners [44, 45]. Some studies indicate that zero tolerance approach to alcohol may delay alcohol use initiation thus reducing alcohol related harm, however, once alcohol use becomes more prevalent during adolescents the abstinence messaging becomes less effective and should be supplemented by harm minimization approaches [46, 47]. Harm reduction programs focus on minimizing the severe consequences of alcohol use, and regard circumstances in which alcohol is consumed to be the main culprit of severe outcomes of alcohol use [48, 49]. Furthermore, other studies suggest that zero tolerance interventions are unsuccessful in limiting alcohol consumption and may even increase alcohol use during adolescence [50, 51]. Adolescents are often more skeptical to institutional control and have higher tendencies to rebel, thus being more likely to challenge societal norms and rules including alcohol use [50, 52]. Although, some abstinent adolescents attribute their attitude to the zero-tolerance approach, the effect of this type of rhetoric may be limited to those individuals who are inherently less likely to initiate alcohol use due to their personal predisposition [53].

The setting of our study poses an additional challenge to the teachers’ zero-tolerance approach. Both teachers and learners reported that alcohol was readily available, consumed in the community, and difficult to avoid. Previous studies also indicate that children were exposed to daily alcohol use by their parents, the community, and through commercials [6, 8, 11]. Alcohol can be served in proximity of schools and at times free sampling can be offered to children [6, 11]. Considering the wide availability of alcohol and environments conducive to drinking, the zero tolerance- and alcohol avoidance-approaches appear incongruent with the daily reality seen and experienced by school-attending children in Eastern Uganda.

#### Avoidance of peers

In this study participants attributed peer pressure as the main reason for initiation and consumption of alcohol. This is confirmed by a study from Ugandan schools that indicated peer influence as one of the main factors for alcohol and substance use in schools [54]. Although, there is a common assumption linking peers to alcohol use the evidence is mixed regarding the causality between peer influence and substance use [55]. Some studies suggest a causal relationship, while others propose that adolescents may seek out like-minded friends, after initiating substance use, or that behaviors evolve together within the friend group [16, 42, 55]. Generally, most young people drink for enhanced enjoyment in social gatherings and these enhancement motives are associated with personality traits of sensation seeking and with low inhibitory control [56–58]. Overall, “Say No!” programs targeting refusal skills and resistance to peer influence have shown little effect compared to programs that target social norms, passive social pressure and interpersonal skills [55].

#### Danger focused rhetoric

Universal alcohol curriculum should include several key elements: dispelling misconceptions of substance use, including prevalence and the positive and negative effects of alcohol, addressing children’s perceptions of the risks associated with substance use and providing life-skills on alcohol decision making [40]. In this study, teachers emphasized teaching children the negative consequences of substance use. Consequently, learners showed extensive knowledge of the harmful effects of alcohol. Teachers reasoned that knowledge of these negative effects would prevent children from consuming alcohol. One study from Tanzania supports this notion indicating that fear of side effects was one of the reasons for abstaining from alcohol [57]. Although teachers from our study focused on the severe biological, mental, and social consequences of harmful alcohol use, they also regarded these dangers to be realistic for their community. Similarly, studies report that Ugandan children face high rates of sexual and physical violence as well as homelessness, lack of basic needs and social support where alcohol use can be one of the contributing factors for these negative outcomes [6, 11, 59, 60]. Thus, while the alcohol use dangers taught by the teachers were severe, they were also grounded in reality of Ugandan children.

However, the aim of teaching these negative effects of alcohol use was to scare children rather than provide them with factual and nuanced information of the negative effects of substance use. One review showed that in some instances scare tactics could be beneficial and reduce substance use, however, they pointed out that the methods used should not be moralizing, exaggerated or raise demands for zero substance use [61]. Overall, focusing only on emotional education and fear arousal is considered ineffective alcohol use prevention method [40]. The moralizing and didactic nature of zero tolerance and scare tactics may hinder open discussion on alcohol use. Furthermore, stigmatizing alcohol users as criminals and nuisance to society and labeling children as “spoiled” and troubled may alienate struggling learners and hinder them from seeking help. Additionally, since teachers focused most on the unacceptable uses of alcohol and had zero tolerance approach to drinking, less attention was given to addressing responsible alcohol use including alcohol use attitudes such as not pressuring others to drink and behavior, how much people drink and how often. We noticed that children had varying opinions on acceptable amounts and frequency of alcohol use and teachers provided little nuance on how much alcohol should be consumed for given effects to occur.

Our findings suggest that teachers regarded alcohol education as an important subject. Many teachers showed concern and great care for their learners and aimed to prevent alcohol use by children. However, the didactic and moralizing methods of teaching alcohol use are not supported by literature. Therefore, a disapproving school standard against alcohol use could be supplemented by a more pragmatic harm reduction approach that acknowledges the prevalence and availability of alcohol in Uganda. Although, from the health perspective zero alcohol consumption is optimal, moral abstinence education is ineffective [40, 61]. Meanwhile, harm reduction programs have shown to be more effective at reducing the negative consequences of alcohol use [50]. Focusing on responsible drinking later in life, and an inclusive environment for younger experimenting peers, may reduce heavy drinking episodes and limit the related problems [49]. Furthermore, a harm reduction approach may especially benefit learners who have already tested alcohol and may lead to less stigma and marginalization of these children. Overall, the use of zero tolerance and danger rhetoric approaches do not reflect the realities the Ugandan children face daily and do not equip them with tools to prevent harm from alcohol consumption [53]. The legal drinking age in Uganda is 18, but many children consume earlier [11, 13, 27, 62]. The schools could prepare learners with more nuanced and age-appropriate information and contribute to reduced harm.

### Strengths and limitations

By using different data sources, we were able to capture the alcohol education from both the learners’ and teachers’ perspectives. Through purposive sampling we included schools with different characteristics, thus the data was collected from a broad demographic spectrum of participants and captured a wider range of opinions. The two Ugandan research assistants moderated the FGDs in the preferred language by the children to improve cultural sensitivity. The same researchers also transcribed and translated the data. To minimize cultural bias of the first author, she worked closely with local data collectors and was advised by a Ugandan supervisor (JNB). Furthermore, the results were validated within the wider research group, Ugandan national stakeholders and with 6 teachers who participated in the interviews.

We stratified the focus groups by gender to increase homogeneity within the group to facilitate a favourable group dynamic. The groups were not divided by age nor grade which may have influenced the group dynamic possibly silencing the younger children. Since learners were selected from the same school for each FGD, the group dynamic and psychological safety of the participants may also have been influenced by existing peer and social structures. We note that not all learners aged 11–13 years had completed grade 6 and their experiences were therefore limited to the curriculum they had been taught. We accepted this because we preferred to obtain a more diverse group of participants. Collaborating with the teachers was deemed appropriate as they were familiar with the children, their parents, and the community, thus facilitating access to the study participants. We acknowledge that this approach is prone to selections bias because teachers may have selected favored and best achieving learners within the eligible population. Thus, this study may not have captured the most vulnerable population of primary school learners. However, this approach to sampling ensured access to the participants and selection of children who were comfortable with expressing themselves. Furthermore, some learners perceived the discussion as an evaluation of their knowledge rather than an inquiry into their experiences and opinions. Likewise, some teachers were reluctant to talk about alcohol issues within their school, opting instead to give examples from previous employment or other schools in the area. We suspect that conducting interviews and discussions on school premises during school hours may have caused participants to be more constrained. Lastly, we recognize that due to using translated FGD transcripts we compromised original expressions and concepts. To mitigate this, the same moderators transcribed and translated the data to maintain its integrity.

#### Implications

This study explored the teachers’ and learners’ perspectives on alcohol education. Factors such as improved student-teacher ratio, stronger collaboration between teachers and the health sector and improved teacher skills with counselling can all benefit the health of the children. Studies suggest that out of school youth in Uganda consume more alcohol, thus, better retention rates in school and interventions on alcohol are important for improving health in Uganda [63]. Furthermore, effective alcohol policies need to be implemented to support alcohol education in schools, limiting the availability and promotion of alcohol, especially targeting children and youth [2, 9]. Lastly, more research on child alcohol use and effective school-based alcohol interventions are needed in Africa and Uganda.

## Conclusion

This study shows that teachers considered alcohol education as an important topic aiming to teach children about the dangers and harmful effects of alcohol consumption. Teachers participating in this study followed the science curriculum teaching four principal aspects of alcohol education: production of alcohol, reasons for taking alcohol, effects of alcohol, and how to avoid it. They employed two main strategies to discourage children from drinking: avoidance of places and peers that consumed alcohol as well as the danger-focused rhetoric aiming to provide knowledge about the dangers of alcohol and foster negative attitudes towards it. Similarly, the learners expressed negative attitudes towards alcohol, emphasizing its harmful effects on the body, community, family and their peers.

## Electronic supplementary material

Below is the link to the electronic supplementary material.

Supplementary Material 1

Supplementary Material 2

## Data Availability

The University of Bergen and Makerere University have shared intellectual property rights to the data. The datasets used and analyzed during the current study are available from the corresponding author (NV) on reasonable request and necessary approvements.

## Abbreviations

FGD: Focus group discussion
KII: Key informant interview
