# Supplementary material for: Alcohol education in Ugandan primary schools: teaching approaches and learners’ perspectives
Source: BMC Public Health. 2025 May 17;25:1823. doi: 10.1186/s12889-025-23043-1 (PMC12084952; doi:10.1186/s12889-025-23043-1)
Supplement: Supplementary file 1 — Supplementary Material 1 [file 12889_2025_23043_MOESM1_ESM.pdf]

Appendix 1: Key Informant Interviews with the Teachers

Variables to note

School's name:<br><br>School's classification:<br><br>Education level of the participant:<br><br>Subjects taught by the participant:<br><br>Years of experience:<br><br>Years of teaching health classes/nature science classes:<br><br>Participant's age:<br><br>Participant's gender:<br><br>Participant's contact information:<br><br>- Name<br>- Email<br>- Phone number<br><br>Code number given to the participant:

Quick discerption of how the KII went. Strengths and challenges during the discussion. Other noteworthy comments.

Preliminary topic guide for key informant interviews with teachers

Thank you very much for coming here today to talk to us about health education and the topics of mental health, alcohol and substance use. This project is a collaboration between Makarere university and University of Bergen in Norway.

My name is Neda and I am a medical research student from Norway. Together with me I have… My role as moderator will be to guide the discussion and my colleague will be taking notes throughout our interview.

We have invited you to take part in this study because we are very interested in your experiences with teaching health classes here in Mbale. We are here to learn from you and to better understand your opinions on this matter. Your positive, negative comments and experiences are welcome, so please share all your thoughts and opinions.

Before we start. You have previously been given an information sheet about this study. We would like to inform you once more that we will be using an audio recorder to record this discussion. We're recording the session because we don't want to miss any of the information you provide us with. People often say very helpful things in these discussions, and we can't write fast enough to get them all down. Are you ok with that?

Teachers' views on provided health education.

Intro: First we would like to ask you some questions about health education in general. When I say health education, I mean classes you teach about health, body, mind, including: hygiene, nutrition, puberty, physical exercise, mental health, substance and alcohol use.

Questions:

1. How did you become health education teacher?
   - Did you receive any specific training/education relating to health? If so, what kind?
2. Could you tell me about the health education programme in your school?
   - In your opinion, are there any topics that are not covered by the health education curriculum? Why?
   - In your opinion, are there any existing topics in the curriculum that should get more attention? Why?

3. Could you take me through any of the activities that you do during health education classes?

4. What kinds of teaching methods do you use during health classes?
   - Which methods are the most effective and why?

5. How much priority does your school give to health education?

6. Which internal factors influence your provision of health education? Which external factors influence your provision of health education?

   - Could you mention any benefits of providing health education in your school?

DO NOT COPY

- What kind of challenges do you face in providing health education?
- What recommendations do you have for dealing with these challenges?

Teachers' views on health.

Intro: Next I would like to ask you some questions regarding health and your opinions on health of the school going children?

7. What are the biggest challenges facing youths today when it comes to health?

- How do these health challenges affect pupils education?

8. What role does the school have in promoting health?

- In your opinion what role should the school have in health promotion?

Mental health

9. Tell me about …. What children learn about mental health in schools today,

- How do children benefit from provided mental health education?
- What should be improved about mental health education
- How can this be done?

Teachers' thoughts on alcohol and substance use

10. During health classes, what do you teach your pupils about alcohol and substance use? If nothing: In your opinion, what should you teach your pupils about alcohol and substance use?

11. Have you noticed any pupils consuming alcohol or using substance?

- What did you do when you noticed a pupil consuming alcohol or using other substances?
- If: nothing what would you have liked to do?

12. What would the school do if you suspected that a student has mental, cognitive or behavioural difficulties?

DO NOT COPY
