# Supplementary material for: Alcohol education in Ugandan primary schools: teaching approaches and learners’ perspectives
Source: BMC Public Health. 2025 May 17;25:1823. doi: 10.1186/s12889-025-23043-1 (PMC12084952; doi:10.1186/s12889-025-23043-1)
Supplement: Supplementary file 2 — Supplementary Material 2 [file 12889_2025_23043_MOESM2_ESM.pdf]

Appendix 2: Focus Group Discussions with Preadolescents

Variables to note

Schools' name:

Schools' classification:

Grade of the participants:

Number of participants:

Gender of participants:

Age of the participants:

Participants' names and code numbers

1.

2.

3.

4.

5.

Quick discerption of how the FGD went. Strengths and challenges during the discussion. Other noteworthy comments.

Focus group discussion guide with preadolescents

Welcome

Thank you very much for coming here today to talk to us about health education and the topics of mental health, alcohol and substance use. My name is… (Introduction of the moderator and the assistant). You are invited to take part in this study because we are very interested in your experiences and opinions on these topics. There are no right or wrong answers, only different points of view. Please, feel free to share your point of view even if it is different from what others have said. You don't need to agree with others, but you must listen respectfully as others share their thoughts. We're just as interested in your negative comments as positive comments so please share all your thought and opinions. My role as moderator will be to guide the discussion. The goal of this discussion is that you talk to each other, share your experiences and thoughts with one another. Before we start. You have previously been given an information sheet about this study. We would like to inform you once more that we will be using audio recorder to record this discussion. We're tape recording the session because we don't want to miss any of your comments. People often say very helpful things in these discussions, and we can't write fast enough to get them all down. Is everyone ok with that?

Views on provided health education.

Intro: We now want to talk to you about health education in your school. When we say health education we mean: information you received or learned about health, body, mind, including: hygiene, nutrition, puberty, physical exercise, mental health, substance and alcohol use.

Questions:

1. Think back to the health classes you have had in school. What did you learn about?
   - Which topics did you find interesting or useful? Why?
   - Which topics did you find boring? Why?

2.Which other topics do you think should be taught during health classes?

3. If you are the one in charge, how would you make health classes better?

   - What could the teachers do better?
   - How could the teacher make health classes more engaging?

Preadolescents' opinions on health

Intro: Next, we would like to discuss health and what health means to you

Questions:

4. What do you think makes a person healthy?

   - How can a person maintain good health?

5. What are the biggest challenges facing youths today when it comes to health?

DO NOT COPY

- What do you and your friends worry about when it comes to your own health?

Preadolescents' thoughts on mental health

Intro: when we talk about health we often talk about physical wellbeing, such as not being sick. Now we would like to talk about being healthy in your mind and having good mental health.

Questions:

6. What do you know about mental health

- Where have you learned this? From school or elsewhere?

7. How do you think your feelings and thoughts can affect your health?

- Which emotions lead to bad health? Why?
- Which emotions lead to good health? Why?

8. Who would you talk to if you were having difficult thoughts and emotions?

- How could this person/these persons help you?

9. How can health classes help youths with mental health?

Preadolescents' thoughts on alcohol and substance use

Intro: Next, we would like to ask about your thoughts on alcohol and substance use. When we say alcohol, we mean drinking alcohol such as beer, waragi or other alcoholic beverages. When we ask about substance use we mean substances that can affect your brain and function, substances such as cannabis.

Questions:

10. What do you know about alcohol?

- Where have you learned about alcohol?

11. Why do people drink alcohol?H

- What benefits are there for drinking alcohol?
- How does alcohol affect a person's mood?
- How much and how often should a person drink alcohol?

12. How can alcohol and substance use affect someone's health?

- How can alcohol and substance use affect someone's mental health?
- How can alcohol and substance use affect someone's social or everyday life?

13. How would you feel if your friend started drinking alcohol or using other substances?

14. What would you do if your friend started drinking alcohol or using other substances?

15. What would you do yourself if you had problems with alcohol or substance use?

DO NOT COPY
